# Budget impact from the incorporation of positron emission tomography – computed tomography for staging lung cancers

**DOI:** 10.1590/S0034-8910.2015049005447

**Published:** 2015-09-10

**Authors:** Aline Navega Biz, Rosângela Caetano

**Affiliations:** I Programa de Pós-graduação em Saúde Coletiva. Instituto de Medicina Social. Universidade do Estado do Rio de Janeiro. Rio de Janeiro, RJ, Brasil; II Departamento de Planejamento e Administração em Saúde. Instituto de Medicina Social. Universidade do Estado do Rio de Janeiro. Rio de Janeiro, RJ, Brasil

**Keywords:** Positron-Emission Tomography, economics, Carcinoma, Non-Small-Cell Lung, therapy, Health Care Costs, Budgets, Unified Health System

## Abstract

**OBJECTIVE:**

To estimate the budget impact from the incorporation of positron emission tomography (PET) in mediastinal and distant staging of non-small cell lung cancer.

**METHODS:**

The estimates were calculated by the epidemiological method for years 2014 to 2018. Nation-wide data were used about the incidence; data on distribution of the disease´s prevalence and on the technologies’ accuracy were from the literature; data regarding involved costs were taken from a micro-costing study and from Brazilian Unified Health System (SUS) database. Two strategies for using PET were analyzed: the offer to all newly-diagnosed patients, and the restricted offer to the ones who had negative results in previous computed tomography (CT) exams. Univariate and extreme scenarios sensitivity analyses were conducted to evaluate the influence from sources of uncertainties in the parameters used.

**RESULTS:**

The incorporation of PET-CT in SUS would imply the need for additional resources of 158.1 BRL (98.2 USD) million for the restricted offer and 202.7 BRL (125.9 USD) million for the inclusive offer in five years, with a difference of 44.6 BRL (27.7 USD) million between the two offer strategies within that period. In absolute terms, the total budget impact from its incorporation in SUS, in five years, would be 555 BRL (345 USD) and 600 BRL (372.8 USD) million, respectively. The costs from the PET-CT procedure were the most influential parameter in the results. In the most optimistic scenario, the additional budget impact would be reduced to 86.9 BRL (54 USD) and 103.8 BRL (64.5 USD) million, considering PET-CT for negative CT and PET-CT for all, respectively.

**CONCLUSIONS:**

The incorporation of PET in the clinical staging of non-small cell lung cancer seems to be financially feasible considering the high budget of the Brazilian Ministry of Health. The potential reduction in the number of unnecessary surgeries may cause the available resources to be more efficiently allocated.

## INTRODUCTION

Economic evaluation of diagnostic and therapeutic interventions is gaining importance to support decisions concerning the incorporation and dissemination of new health care technologies.^22^ Those analyses, however, do not provide all necessary information for decision-making, as they do not assess the feasibility for the introduction of the best alternative considering available budgets.^11^ The further conduction of budget impact analyses to evaluate short and medium-term financial consequences regarding the incorporation, changed use, or withdrawal of a technology from the set of available interventions in the health care system is required.^2^ ^,^ ^8^

Brazil reports a high number of lung cancer cases: 27,330 new cases are estimated for 2014.^a^ Non-small cell lung carcinomas (NSCLC) account for 75.0%-85.0% of cases, which can be potentially cured with surgical resection in the localized disease.^5^ ^,^ ^b^ Often, the diagnosis is achieved in advanced stages. Thus, due to the disease spread to mediastinal lymph nodes or distant metastases at the time of diagnosis, only 20.0% of patients are considered operable.^c^

Evaluating the disease extension at the diagnosis is essential for defining therapies. That avoids improper procedures which can influence patients’ survival and quality of life. The clinical staging is mainly conducted by means of computed tomography of the thorax and upper abdomen (CT of thorax), according to the clinical guidelines for the diagnosis and treatment of lung cancer, as disclosed by the Brazilian Ministry of Health (MH) in 2012.^d^ That exam is mainly based in morphological changes.

Positron emission tomography (PET) which is either combined to computed tomography (PET-CT) or not, is based on metabolic activity, rather than only on anatomical aspects. Both are more accurate than conventional imaging techniques in the evaluation of mediastinal and in distant areas involvement.^5^ Its inclusion in the traditional diagnostic strategies may result in better management of cases, with reduced numbers of unnecessary surgeries^21^ ^,^ ^23^ and decreased morbidity and mortality. Another advantage would be staging the lung disease and distant metastases with a single exam.^14^

PET is starting to be disseminated in Brazil, and it was included in the Brazilian Unified Health System (SUS) payrolls for procedures in April 2014.^e^ The economic evaluation for the use of PET-CT in the staging of NSCLC, conducted for the MH in 2013, found that PET-CT is more cost-effective when compared to the currently offered management strategy, which is CT-based.^b^ The results confirm international findings,^4^ which show benefits in its inclusion for the staging of NSCLC patients, mainly for preventing unnecessary surgeries, that pay off for the additional costs for using of the new technology.

The study from 2013 did not evaluate the financial impacts from offering the procedure in Brazil’s public health care service network. Budget impact analyses are scarce in Brazil, especially concerning diagnostic imaging. In a health care system which is set to offer universal and comprehensive care, the concern with using resources is shown to be important considering the dichotomic relationship among budget availability, extension of care, and continuous advancements in technology.

This study aimed to estimate the budget impact of the inclusion of PET-CT in the mediastinal and distant staging of non-small cell lung cancer.

## METHODS

The budget impact estimation has adopted SUS’s perspective as a financing agent of health care services, as indicated by the Brazilian guideline.^f^

The chosen horizon was a five-year one (2014 to 2018), considering the possible morosity in the reallocation of government budgets and restrictions in the availability and access to PET-CT.

The projected use of PET-CT was conducted under the epidemiological method. Eligible patients correspond to all newly-diagnosed cases. Thus, estimates for numbers of lung cancer cases for 2006 to 2014 were used, as disclosed by the Brazilian National Cancer Institute (INCA) (Table 1), with 85.0% of total cases assigned to NSCLC histological type.^5^ The number of new cases were estimated by admitting a 75.0% coverage for the SUS-supported patient population.^g^

**Table 1:** Cases of lung cancer and NSCLC, from 2006 to 2014, and the projected number of new NSCLC cases, from 2006 to 2014, and the ones handled by the Brazilian Unified Health System, from 2014 to 2018.

Year	New lung cancer cases	New NSCLC cases	New NSCLC cases handled by SUS
2006	27,170	23,095	–
2007	27,170	23,095	–
2008	27,270	23,180	–
2009	27,270	23,180	–
2010	27,630	23,486	–
2011	27,630	23,486	–
2012	27,320	23,222	–
2013	27,320	23,222	–
2014^a^	27,330	23,231	17,423
2015^b^	–	23,248	17,436
2016^b^	–	23,266	17,449
2017^b^	–	23,283	17,462
2018^b^	–	23,301	17,475

Source: Estimates from the data regarding number of cancer cases, as disclosed by the Brazilian National Cancer Institute in 2005, 2007, 2009, 2011, and 2014.

NSCLC: Non-small cell lung cancer; SUS: *Sistema Único de Saúde* (Brazilian Unified Health System)

a The preliminary data from the following source were repeated: Ministério da Saúde. Instituto Nacional de Câncer. Estimativa 2014: incidência de câncer no Brasil. Rio de Janeiro (RJ); 2014.

b Estimated from the variation regarding years 2006 to 2014.

Three analysis scenarios were defined: reference (strategies of management that are widely used, based on CT of thorax for all patients); alternative 1 (use of PET-CT restricted to patients with previous negative CT results, allowing for coverage of situations with more limited access to PET-CT); alternative 2 (use of CT and PET-CT for all cases, with further clinical management being defined by the combined results of the two exams – only patients with both negative images would directly proceed to pulmonary resection). This last strategy yielded a higher reduction in the number of unnecessary surgeries in the cost-effective study used as basis,^b^ with small differences in the incremental cost-effectiveness ratio between the two usage methods for PET-CT in the conducted sensitivity analyses.

Only direct costs of procedures involved in the staging and therapies of patients were considered (Table 2). As the PET-CT procedure was not included in SUS payrolls when the analyses were conducted, we used values as estimated by micro-costing.^3^ The values were calculated again to have a 30.0% reduction in the F18-fluoro-2-deoxy-D-glucose costs (^18^FDG),^h^ to consider the recent increase in the number of private input producers which took place when the Federal Government lost its monopoly for radiopharmaceuticals in 2006. For all procedures figuring in SUS payroll charts, values regarding November 2013 were used, which were listed in SUS Management System for the Chart of Procedures, Medications, and Orthoses, Prosthetics, and Special Materials.^i^

**Table 2:** Cost parameters (in PPP-adjusted US$, as per 2013 rates)*, accuracy, and epidemiological data that were used in the budget impact analysis and data source.

Parameter	Value	Range	References
Costs*			
CT of thorax (US$)	84.73	–	Sigtap/DataSUS^i^
Whole body PET-CT (US$)	1,662.58	1,017.31;1,818.13	Caetano^3^ (2014) + Premises
Mediastinoscopy (US$)	860.37	–	Sigtap/DataSUS^i^
Biopsy (US$)	598.68	–	Sigtap/DataSUS^i^
Surgery (US$)	2,687.32	–	Sigtap/DataSUS^i^
Chemotherapy + Radiotherapy (US$)	2,416.15	–	Sigtap/DataSUS^i^
Palliative care (US$)	683.23	–	Sigtap/DataSUS^i^
Deaths from mediastinoscopy (US$)	1,687.77	–	Sigtap/DataSUS^i^
Accuracy			
CT of thorax sensitivity for mediastinal lymph nodes (%)	51	47;62	Dwamena^7^ (1999); Silvestri^18^ (2007)
Biopsy sensitivity for distant metastases (%)	100	80;100	Gambhir^9^ (1996); Sloka^19^ (2004)

Epidemiological parameters			

Variation rate of number of cases (%)	0.0754	-1.1983;0.4054	Estimates from INCA in 2005, 2007, 2009, 2011, and 2014
Prevalence of distant metastases (%)	20	12;25	NICE 2011^c^
Prevalence of metastases in mediastinal lymph nodes (%)	30	15;40	Dietlein^6^ (2000); NICE 2011^c^
Probability for conduction of mediastinoscopy (%)	50	0;100	Alzahouri^1^ (2005) refers to specialists

CT: Computed tomography; INCA: Brazilian National Cancer Institute; PET-CT: Positron emission tomography along with computed tomography

* World Bank’s PPP conversion rate for 2013 (PPP-adjusted USD): 1 USD = 1.61 BRL.

For the budget impact estimates, the same decision trees and parameters that were used in the cost-effectiveness study, conducted for MH in 2013, were used again here.^b^ The new cases projected for each year and the costs of procedures fed the trees related to each analysis scenario, which generated estimates for quantities of conducted procedures and total costs associated to that target population. The yearly budget impacts and the budgets for the period between 2014 and 2018 were calculated for each scenario. No discounts rates or values regarding adjust for inflation were introduced, in compliance to international^1^ ^2^ ^,^ ^13^ ^,^ ^16^ and national^f^ guidelines for this type of study.

The incremental budget impact for each examined year was calculated by means of the difference between the total budget impacts for the alternative and reference scenarios. The incremental difference among the alternative strategies was evaluated, which enabled the analysis of a wider and more restricted offer of technology.

Univariate and extreme scenarios sensitivity analyses were conducted to consider the uncertainties related to parameter values and premises used.^15^ The evaluated parameters in the first ones were: the annual variation rate of lung cancer cases; costs of PET-CT procedure; prevalence of mediastinal and distant lesions; probability of conducting confirmatory mediastinoscopy; and CT and PET-CT sensitivity. The same ranges of values that were obtained in the literature and used in the study for the MH were used here.^b^

The parameters were simultaneously modified in the extreme scenarios sensitivity analysis. The “best-case scenario” corresponded to minimizing the budget impact from PET incorporation for any alternative scenario adopted. The minimum values in the range that figures in Table 2 for the following parameter were employed: costs of PET-CT, annual variation rate for the number of new cases and CT sensitivity. Simultaneously, the following were employed considering their maximum values: biopsy sensitivity, share of patients having undergone mediastinoscopy procedure; and prevalence of metastases in mediastinal lymph nodes (N2/3) and distant metastases (M1). The “worst-case scenario” corresponded to the same parameters varying in the opposite direction to the one mentioned above.

Moreover, the influence from the rate by which the technology is disseminated at SUS was analyzed. It is possible that, even with it being included in SUS payrolls, delays may take place until it is fully offered, due to the current geographical availability of equipment and qualified staff for its operation. Sixty percent of patients were considered eligible for using PET-CT in 2014, with 10.0% increases with each year, until full access was achieved in 2018.

Written authorization was obtained from the Project (CNPq 564797/2010-3) coordinator, concerning the usage of data and model of the cost-effectiveness study.

## RESULTS

The current diagnostic and therapeutic management model for NSCLC patients in Brazilian health care services, which is focused on CT use, would result in 397.5 BRL (246.9 USD) million in expenditures^j^ in five years for SUS*.*

The introduction of PET-CT in NSCLC staging would imply an increase in total expenditures for SUS (Table 3) due to its complementary, non-replaceable nature, regardless of the strategy for its use. Its restricted use in patients with negative CT of thorax results would determine a total impact of 555.5 BRL (345.0 USD) million over the period (+39.8% as compared to the current management). Its use for all patients would cause an impact of 600.1 BRL (372.8 USD) million (+51.0%).

**Table 3:** Total and incremental budget impact per year and for 2014 to 2018, regarding the studied analysis scenarios (in PPP-adjusted USD from 2013)a.

Period	Total budget impact^a^			Incremental budget impact^a^		

	CT	PET-CT for CT-^b^	PET-CT for all^c^	PET-CT for CT-^b^ regarding CT	PET-CT for all^c^ regarding CT	PET-CT for all^c^ regarding PET-CT for CT-^b^
2014	49,297,708.43	68,902,114.70	74,438,876.78	19,604,406.27	25,141,168.35	5,536,762.07
2015	49,334,855.45	68,954,034.11	74,494,968.27	19,619,178.66	25,160,112.83	5,540,934.16
2016	49,372,030.45	69,005,992.65	74,551,102.03	19,633,962.19	25,179,071.58	5,545,109.39
2017	49,409,233.47	69,057,990.34	74,607,278.09	19,648,756.86	25,198,044.61	5,549,287.76
2018	49,446,464.53	69,110,027.20	74,663,496.48	19,663,562.67	25,217,031.96	5,553,469.28
2014-2018	246,860,292.33	345,030,158.99	372,755,721.65	98,169,866.66	125,895,429.32	27,725,562.65

CT: Computed tomography; PET-CT: Positron emission tomography along with computed tomography

a World Bank’s PPP conversion rate for 2013 (PPP-adjusted USD for 2013): 1 USD = 1.61 BRL.

b PET-CT for CT-: conduction of PET-CT only for patients with negative CT results.

c PET-CT for all: conduction of PET-CT for all patients, considering both the results from PET and CT for resuming the clinical, therapeutic management.

The financial impact from the more restricted PET-CT offer would imply an additional allocation of 158 BRL (98.2 USD) million in five years (Table 3). Extending the offer to all potential candidates would involve 202.7 BRL (125.9 USD) million in additional resources, with 44.6 BRL (27.7 USD) million being the difference between the strategies at the end of the period.

The cost of PET-CT procedure was the parameter with the biggest impact in the univariate sensitivity analyses (Figure) using the values from the range in Table 2. The reduction in the cost of the procedure to 1,637.87 BRL (1,017.31 USD) would cause a total five-year budget impact reduction of BRL 67.9 (42.2 USD) million in the restricted offer (-12.2%) and 90.6 BRL (56.3 USD) million in the most inclusive use (-15.1%). The difference from the two strategies would drop to 21.9 BRL (13.6 USD) million. An increase in the cost of the procedure to 2,927.19 BRL (1,818.13 USD) would result in relatively smaller increases in the total budget impact: 16.4 BRL (10.2 USD) million (+2.9%) in the restricted offer and 21.9 BRL (13.6 USD) million (+3.6%) in the inclusive offer.

**Figure:**
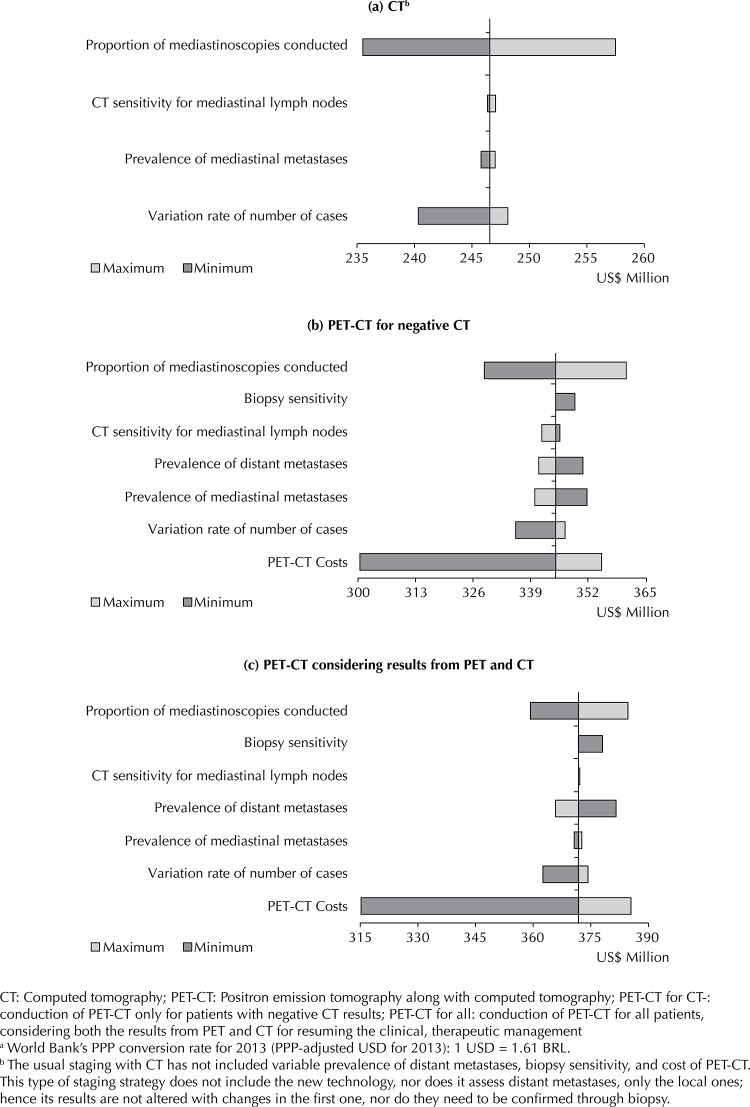
Result from the total budget impact univariate sensitivity analysis (in PPP-adjusted dollars from 2013).a Evaluated scenarios, Brazil, 2014 to 2018.

The variation in the share of patients submitted to mediastinoscopy to confirm imaging exam results, between 0% and 100%, was shown to be important, given their costs to SUS. Non-performance of mediastinoscopy corresponded to a reduction in the total budget impact of 24.6 BRL (15.3 USD) million in the “PET-CT for CT-” scenario, and 20.3 BRL (12.6 USD) million in the use of “PET for all”. Its conduction in all patients, on the other hand, would lead to increases in both scenarios of the same amounts mentioned above.

The use of the lower value of the range of the growth of staging-eligible NSCLC cases produced decreases in the budget impact regardless of the analyzed scenario: from 14 BRL (8.7 USD) million, in the “PET-CT for CT-” scenario, and 15.1 BRL (9.4 USD) million, with the offer of “PET-CT for all”. Using the upper limit of that parameter resulted in increases of 3.7 BRL (2.3 USD) million and 4 BRL (2.5 USD) million, respectively.

The extreme scenarios sensitivity analyses (Table 4) showed significant reduction in total budget impact in the “best-case scenario”: 90.3 BRL (56.1 USD) million in the inclusive use of PET-CT (-15.0%), and 62.5 BRL (38.8 USD) in the restricted offer (-11.3%). The incorporation would result in increased budget impacts of 25.9 BRL (16.1 USD) million in the “PET-CT for all” strategy (+4.3%), and 22.4 BRL (13.9 USD) million in the restricted use (+4.0%) in the “worst-case scenario”.

**Table 4:** Total and incremental budget impact analyses of staging strategies per projected year (in PPP-adjusted USD from 2013)a. Brazil, 2014 to 2018.

Period	Total budget impact^a^			Incremental budget impact^a^		

	CT	PET for CT-^b^	PET-CT for all^c^	PET for CT-^b^ regarding CT	PET-CT for all^c^ regarding CT	PET-CT for all^c^ regarding PET for CT-^b^
Base case						
2014	49,297,708.43	68,902,114.70	74,438,876.78	19,604,406.27	25,141,168.35	5,536,762.07
2015	49,334,855.45	68,954,034.11	74,494,968.27	19,619,178.66	25,160,112.83	5,540,934.16
2016	49,372,030.45	69,005,992.65	74,551,102.03	19,633,962.19	25,179,071.58	5,545,109.39
2017	49,409,233.47	69,057,990.34	74,607,278.09	19,648,756.86	25,198,044.61	5,549,287.76
2018	49,446,464.53	69,110,027.20	74,663,496.48	19,663,562.67	25,217,031.96	5,553,469.28
2014-2018	246,860,292.33	345,030,158.99	372,755,721.65	98,169,866.66	125,895,429.32	27,725,562.65
Best-case scenario^d^						
2014	51,669,368.28	62,721,624.22	64,875,377.64	11,052,256.14	13,206,009.32	2,153,753.42
2015	51,050,206.32	61,970,021.74	64,097,965.84	10,919,815.26	13,047,759.63	2,127,944.72
2016	50,438,463.86	61,227,425.47	63,329,870.19	10,788,961.44	12,891,406.21	2,102,445.34
2017	49,834,052.01	60,493,727.95	62,570,978.88	10,659,675.66	12,736,926.71	2,077,250.93
2018	49,236,882.92	59,768,821.74	61,821,181.37	10,531,939.13	12,584,298.14	2,052,359.01
2014-2018	252,228,973.39	306,181,621.01	316,695,374.12	53,952,647.63	64,466,400.74	10,513,753.11
Worst-case scenario^e^						
2014	47,295,407.04	71,202,595.65	77,144,317.39	23,907,188.32	29,848,910.56	5,941,721.74
2015	47,487,161.13	71,491,278.26	77,457,090.68	24,004,117.42	29,969,929.19	5,965,811.80
2016	47,679,692.66	71,781,132.30	77,771,131.68	24,101,439.50	30,091,439.13	5,990,000.00
2017	47,873,004.79	72,072,160.87	78,086,446.58	24,199,156.16	30,213,441.61	6,014,285.71
2018	48,067,100.68	72,364,369.57	78,403,039.75	24,297,269.01	30,335,939.13	6,038,669.57
2014-2018	238,402,366.32	358,911,536.71	388,862,025.90	120,509,170.40	150,459,659.58	29,950,489.18

CT: Computed tomography; PET-CT: Positron emission tomography along with computed tomography

a World Bank’s PPP conversion rate for 2013 (PPP-adjusted USD for 2013): 1 USD = 1.61 BRL.

b PET-CT for CT-: conduction of PET-CT only for patients with negative CT results.

c PET-CT for all: conduction of PET-CT for all patients, considering both the results from PET and CT for resuming the clinical, therapeutic management.

d Best-case scenario: variation for the lower limit of the parameter interval: cost of PET-CT, annual variation in number of cases, and CT sensitivity; and for maximum values of parameters: biopsy sensitivity, share of patients who were submitted to mediastinoscopy and prevalences of metastases in mediastinal lymph nodes (N2/3) and distant metastases (M1).

e Worst-case scenario: variation for the upper limit of the parameter interval: cost of PET-CT, annual variation in number of cases, and CT sensitivity; and for minimum values of parameters: biopsy sensitivity, share of patients who were submitted to mediastinoscopy and prevalences of N2/3 and M1.

The total reduction in the budget impact would be of 31.6 BRL (19.6 USD) million (-5.7%) in the scenario with the restricted offer of the technology, and 44 BRL (27.4 USD) million with availability to all (-7.3%), considering a progressive dissemination – from 60.0% to 100% in five years – of PET-CT at SUS.

## DISCUSSION

The incorporation of PET-CT in the staging of NSCLC, a highly relevant neoplasia in Brazil’s nosological scenario, would imply total expenditures of 555.5 BRL (345.0 USD) million to SUS, in case its use is restricted to patients with previous negative results in computed tomography of thorax, and 600.1 BRL (372.8 USD) million, in the situation in which it is offered to all new cases that are diagnosed in the period. These values represent additional costs to the current expenditures with computed tomography-based staging, of around 158.1 BRL (98.2 USD) to 202.7 BRL (125.9 USD) million in five years, depending on its more restricted or inclusive use. In absolute or incremental terms, the estimated values reinforce the importance of properly planning and managing of budgets and governmental actions, including health care, in a way to optimize the use of available resources, which are scarce in our field.

The National Policy on Health Technologies Management^k^ and Law 12,401^l^ have recognized the role of complementary economic evaluation. The conduction of budget impact studies to support decisions regarding the incorporation of new technologies at SUS is explicitly recommended.

One of SUS’s challenges lies in its compliance to the principle that health services should follow the principle of universality. Offering PET-CT to all candidates may not be feasible due to financial, infrastructural, or human resources limitations, among others. That acknowledgment, plus the fact that the literature and the study to the Ministry of Health point towards higher health benefits for the group with previous negative CT exams^6^ ^,^ ^b^ led to the simulation of the restricted offer for the exam. However, extending the offer to all potential candidates would result in an increased total budget impact of only 44.6 BRL (27.7 USD) million at the end of the period.

The extent of the impacts which are associated with the incorporation of PET-CT would have significant financial implications, especially if the number of eligible patients were weighted in. That is so because, in 2013, the number of new lung cancer cases corresponded to only 0.01% of the Brazilian population.

The expenditures of the Ministry of Health, which figure in the Annual Budget Act for 2014,^m^ were looked after for better understanding of the meaning of the resources volumes which were estimated with the incorporation of PET-CT. The estimated amount needed to maintain SUS’s current management of the disease corresponds to 0.075% of the 106,019,264,465.00 BRL (65,850,474,822.98 USD) that were predicted for 2014. In the alternative scenarios, the total budget impact estimated would correspond to 0.105% of the MH budget (restricted offer) or to 0.113% (inclusive offer).

Another way to examine how substantial the estimated impacts are would be to compare them to the sums which are spent by SUS with care of lung cancer patients, e.g., related to diagnostics and treatment in their various modalities. However, no consolidated information was found in the literature, nor was it in official documents about expenditures made for that condition. The expenditures from the Ministry of Health that are related to CT of thorax exams, conducted and approved by SUS, can be obtained from the *Sistema de Informações Ambulatoriais* (System of Ambulatorial Information).^n^ But this information correspond to the use of the procedure in several clinical indications (neoplastic or non-neoplastic), and not only for lung cancer, which renders any comparison impossible. The expenditures with inpatient care from SUS related to lung cancer, which were obtained from the *Sistema de Informações Hospitalares* (System of Hospital Information), added up to 23,405,185.25 BRL (14,537,382.14 USD) from January to November 2013.^o^ That makes up for 29.5% of the 79.4 BRL (49.3 USD) million of the budget impact that was estimated in the reference scenario for 2014, but it does not include the remaining diagnostic and therapeutic components which are involved with handling the condition.

The dissemination of PET-CT into the clinical practice took place in a context in which concern with expenditures and impacts for health care systems was building up. Thus, the technology was the subject of several cost-effectiveness studies in several countries. Budget impact evaluations for its implementation are less frequent in the literature, and that is maybe so because they are conducted internally in the governmental environment which is involved with offering the technology. Nonetheless, directly comparing the results of those budget impact analyses with the ones herein is inappropriate. That is so because the management and organization of health care systems, structures of their models, epidemiological data, and especially the underlying cost structures greatly differ among studies.^17^

Comparing budget impact estimates that are conducted in our reality would be ideal. Even though the MH has internally simulated the budget impact from PET at SUS,^p^ its estimation methods and likelihood of bearing important methodological biases hinders comparisons with the results from this study. Besides that, according to the budget impact guidelines, estimates should not be restricted to comparing amounts and prices of technologies per se, but to the financial result from the set of clinical consequences and diagnostic, therapeutic procedures that relate to examined technologies, as this study aimed at.

Although employing PET does not show a significant increase in the survival of patients,^4^ its use allows for better (financial, material, and human) resource distribution in the system, as it more accurately identifies the extension of disease and allows planning the therapeutic strategy that is the most adequate to each case. Such smoother method would prevent unnecessary surgical procedures, which is more relevant when there are famous problems with access to health care services in the country, especially regarding oncology, and significant regional discrepancies in its offer.^10^ ^,^ ^q^

Budget impact studies are scarce, and only more recently they have gained guidelines on good practices more established. This study followed the main available guidelines on budget impact analyses from Task Force on Good Research Practices from the International Society for Pharmacoeconomics and Outcomes Research^13^ and the ones from the Ministry of Health, which were recently published.^f^ Required adaptations were made, as they mainly focus on therapeutic procedures.

Despite our using a nine-year time series (2006 to 2014) to estimate future lung cancer new cases, it was not possible to predict possible alterations arising from population changes or in the prevalence of some of its risk factors. Besides that, this study used parameter values from the cost-effectiveness study. Thus, the same limitations from before remain, as a gap in the national data regarding some epidemiological parameters, accuracy measurements for diagnostic technologies from international studies, and from the missing information about the share of patients who are submitted to mediastinoscopy within the country. The multiple sensitivity analyses conducted aimed at shedding some light on those uncertainties, and potentializing the knowledge regarding the extent of the impact they generate to SUS.

Trueman et al^20^ discuss the incompatibility between the effort to maximize efficiency, which is the core target of economists, and the limits for the current budgets, which is commonly the main need from managers. Budget impact analyses do not show the best way to distribute available resources in the economy, whose most proper evidence come from comprehensive economic evaluation studies, such as the cost-effectiveness ones. Furthermore, the decisions to incorporate technologies in health care systems must take into account other factors, such as the availability of human and budget resources, political factors, and aspects regarding equal access to health care.

Data that is similar to the ones in this study, along with the evidence the technology is cost-effective in Brazil, may allow decisions taken to be properly backed up. Thus, the incorporation of PET in the clinical staging of potentially resectable NSCLC seems to be financially feasible considering the high total budget from Brazil’s Ministry of Health and the potential reduction in the number of unnecessary surgeries better staged patients are submitted to. This may cause the available resources to be more efficiently distributed.

## Footnotes

a Ministério da Saúde. Instituto Nacional de Câncer. Estimativa 2014: incidência de câncer no Brasil. Rio de Janeiro (RJ); 2014.

b Caetano R, Biz AN, Bastos CRG, Garay OU, Schluckebier L. Avaliação econômica: análise de custo-efetividade do uso da 18FDG-PET/TC no estadiamento do câncer pulmonar de células não pequenas: relatório preliminar de pesquisa. Rio de Janeiro (RJ): Instituto de Medicina Social da UERJ; 2013.

c National Collaborating Centre for Cancer. Lung cancer. The diagnosis and treatment of lung cancer. London: NICE; 2011 [cited 2012 Oct 10] (Nice Clinical Guidelines; 121). Available from: http://www.ncbi.nlm.nih.gov/books/NBK99021/

d Ministério da Saúde. Portaria nº 600, de 26 de junho de 2012. Aprova as diretrizes diagnósticas e terapêuticas do câncer de pulmão. *Diário Oficial União.* 28 Jun 2012;Seção1:210.

e Ministério da Saúde. Portaria nº 7, de 22 de abril de 2014. Torna pública a decisão de incorporar o PET-CT no estadiamento clínico do câncer de pulmão de células não-pequenas potencialmente ressecável no Sistema Único de Saúde - SUS. *Diário Oficial União.* 23 Abr 2014;Seção1:78.

f Ministério da Saúde. Secretaria de Ciência, Tecnologia e Insumos Estratégicos. Diretrizes metodológicas: análise de impacto orçamentário: manual para o Sistema de Saúde do Brasil. Brasília (DF); 2012.

g Agência Nacional de Saúde Suplementar. Caderno de informação da Saúde Suplementar: beneficiários, operadoras e planos. Rio de Janeiro (RJ); 2013.

h Ministério da Ciência, Tecnologia e Inovação. Comissão Nacional de Energia Nuclear (CNEN). Relatório de Gestão do Exercício de 2011. Rio de Janeiro (RJ); 2012.

i Ministério da Saúde. Departamento de Informática do SUS (DATASUS). Sistema de Gerenciamento da Tabela de Procedimentos, Medicamentos e OPM do SUS (SIGTAP). Brasília (DF); 2013 [cited 2013 Nov 25]. Available from: http://sigtap.datasus.gov.br/tabela-unificada/app/sec/inicio.jsp

j The conversion of all monetary amounts to the American dollar that is presented in the text, in the tables, and figure, was conducted based on the PPP conversion factor for Brazil, regarding the year of 2013, as disclosed by the World Bank. PPP conversion factor, GDP (LCU per international $). Washington (DC): World Bank; 2015 [cited 2015 May 2]. Available from: http://data.worldbank.org/indicator/PA.NUS.PPP

k Ministério da Saúde. Secretaria de Ciência, Tecnologia e Insumos Estratégicos. Política de Gestão de Tecnologias em saúde. Brasília (DF); 2011.

l Brasil. Lei nº 12.401, de 28 de abril de 2011. Altera a Lei nº 8.080, de 19 de setembro de 1990, para dispor sobre a assistência terapêutica e incorporação de tecnologias em saúde no âmbito do SUS. *Diário Oficial União*. 29 Abr 2011:Seção1:1-2.

m Senado Federal. Portal do Orçamento. Lei Orçamentária Anual 2014: Volume IV –Detalhamento das Ações – Órgão do Poder Executivo – Presidência da República e Ministérios (exceto MEC). 36000. Brasília (DF); 2014 [cited 2014 Jan 13]. Available from: http://www12.senado.gov.br/orcamento/loa?ano=2014&categoria=3.1.7&fase=elaboracao

n Ministério da Saúde. Departamento de Informática do SUS (DATASUS). Sistema de Informações Ambulatoriais do SUS (SIA/SUS). Brasília (DF); 2014. Available from: http://www2.datasus.gov.br/DATASUS/index.php?area=0202

o Ministério da Saúde. Departamento de Informática do SUS (DATASUS). Sistema de Informações Hospitalares do SUS (SIH/SUS). Brasília (DF); 2014 [cited 2014 Jan 15]. Available from: http://www2.datasus.gov.br/DATASUS/index.php?area=0202

p Ministério da Saúde. Secretaria de Ciência, Tecnologia e Insumos Estratégicos. PET-TC no estadiamento de câncer pulmonar de células não-pequenas. Relatório de Recomendação da Comissão Nacional de Incorporação de Tecnologias no SUS – CONITEC-107. Brasília (DF); 2014. Available from: http://portalsaude.saude.gov.br/images/pdf/2014/abril/23/Relatorio-PET-EstadiamentoCancerPulmonar-FINAL.pdf

q Tribunal de Contas da União. Política Nacional de Atenção Oncológica; Relator Ministro José Jorge. Brasília (DF); 2011.

## References

[1] Alzahouri K, Lejeune C, Woronoff-Lemsi MC, Arveux P, Guillemin F (2005). Cost-effectiveness analysis of strategies introducing FDG-PET into the mediastinal staging of non-small-cell lung cancer from the French healthcare system perspective. Clin Radiol.

[2] Brosa M, Gisbert R, Rodríguez JM, Soto J (2005). Principios, métodos y aplicaciones del análisis del impacto presupuestario en el sector sanitario. Pharmaco Econ Spa Res Art.

[3] Caetano R, Schluckebier L, Bastos CRG, Silva RM, Carneiro MP, Silva JWE (2014). Análise dos custos do procedimento PET-TC com 18F-FDG na perspectiva do SUS provedor: estudo em uma unidade pública de saúde do Rio de Janeiro, Brasil. Cad Saude Publica.

[4] Cao JQ, Rodrigues GB, Louie AV, Zaric GS (2012). Systematic review of the cost-effectiveness of Positron-Emission Tomography in staging of non-small-cell lung cancer and management of solitary pulmonary nodules. Clin Lung Cancer.

[5] Devaraj A, Cook GJ, Hansell DM (2007). PET/CT in non-small cell lung cancer staging-promises and problems. Clin Radiol.

[6] Dietlein M, Weber K, Gandjour A, Moka D, Theissen P, Lauterbach KW (2000). Cost-effectiveness of FDG-PET for the management of potentially operable non-small cell lung cancer: priority for a PET based strategy after nodal-negative CT results. Eur J Nucl Med.

[7] Dwamena BA, Sonnad SS, Angobaldo JO, Wahl RL (1999). Metastases from non-small cell lung cancer: mediastinal staging in the 1990s-meta-analytic comparison of PET and CT. Radiology.

[8] Ferreira-da-Silva AL, Ribeiro RA, Santos VCC, Elias FTS, d’Oliveira ALP, Polanczyk CA (2012). Diretriz para análises de impacto orçamentário de tecnologias em saúde no Brasil. Cad Saude Publica.

[9] Gambhir SS, Hoh CK, Phelps ME, Madar I, Maddahi J (1996). Decision tree sensitivity analysis for cost-effectiveness of FDG-PET in the staging and management of non-small-cell lung carcinoma. J Nucl Med.

[10] Gomes SCS, Almeida RT (2009). Modelo de simulação para estimar a infraestrutura necessária à assistência oncológica no Sistema Público de Saúde. Rev Panam Salud Publica.

[11] Hilden J (2008). Budget impact analysis and its rational basis. Med Decis Making.

[12] Marshall DA, Douglas PR, Drummond MF, Torrance GW, Macleod S, Manti O (2008). Guidelines for conducting pharmaceutical budget impact analyses for submission to public drug plans in Canada. Pharmacoeconomics.

[13] Mauskopf JA, Sullivan SD, Annemans L, Caro J, Mullins CD, Nuijten M (2007). Principles of good practice for budget impact analysis: report of the ISPOR Task Force on good research practices - budget impact analysis. Value Health.

[14] Nguyen VH, Peloquin S, Lacasse Y (2005). Cost-effectiveness of positron emission tomography for the management of potentially operable non-small cell lung cancer in Quebec. Can Respir J.

[15] Nuijten MJC, Mittendorf T, Persson U (2011). Practical issues in handling data input and uncertainty in a budget impact analysis. Eur J Health Econ.

[16] Orlewska E, Mierzejewski P (2004). Proposal of Polish guidelines for conducting financial analysis and their comparison to existing guidance on budget impact in other countries. Value Health.

[17] Sculpher MJ, Pang FS, Manca A, Drummond MF, Golder S, Urdahl H (2004). Generalisability in economic evaluation studies in healthcare: a review and case studies. Health Technol Assess.

[18] Silvestri GA, Gould MK, Margolis ML, Tanoue LT, McCrory D, Toloza E (2007). Noninvasive staging of non-small cell lung cancer: ACCP evidenced-based clinical practice guidelines (2nd edition). Chest.

[19] Sloka JS, Hollett PD, Mathews M (2004). Cost-effectiveness of positron emission tomography for non-small cell lung carcinoma in Canada. Med Sci Monit.

[20] Trueman P, Drummond M, Hutton J (2001). Developing guidance for budget impact analysis. Pharmacoeconomics.

[21] van Tinteren H, Hoekstra OS, Smit EF, van den Bergh JH, Schreurs AJ, Stallaert RA (2002). Effectiveness of positron emission tomography in the preoperative assessment of patients with suspected non-small-cell lung cancer: the PLUS multicentre randomised trial. Lancet.

[22] Vianna CMM, Caetano R (2005). Avaliações econômicas como um instrumento no processo de incorporação tecnológica em saúde. Cad Saude Colet.

[23] Viney RC, Boyer MJ, King MT, Kenny PM, Pollicino CA, McLean JM (2004). Randomized controlled trial of the role of positron emission tomography in the management of stage I and II non-small-cell lung cancer. J Clin Oncol.

