# Supplementary material for: Budget impact from the incorporation of positron emission tomography – computed tomography for staging lung cancers
Source: Rev Saude Publica. 2015 Sep 10;49:57. doi: 10.1590/S0034-8910.2015049005447 (PMC4544594; doi:10.1590/S0034-8910.2015049005447)
Supplement: Translation

# Translation: Impacto orçamentário da incorporação da tomografia de emissão de pósitrons – tomografia computadorizada para estadiamento de câncer pulmonar

**Authors:** Aline Navega Biz, Rosângela Caetano

**Affiliations:** I Programa de Pós-graduação em Saúde Coletiva. Instituto de Medicina Social. Universidade do Estado do Rio de Janeiro. Rio de Janeiro, RJ, Brasil; II Departamento de Planejamento e Administração em Saúde. Instituto de Medicina Social. Universidade do Estado do Rio de Janeiro. Rio de Janeiro, RJ, Brasil

**Keywords:** Tomografia por Emissão de Pósitrons, economia, Carcinoma Pulmonar de Células Não Pequenas, terapia, Custos de Cuidados de Saúde, Orçamentos, Sistema Único de Saúde

## Abstract

**OBJETIVO:**

Estimar o impacto orçamentário da incorporação da tomografia por emissão de pósitrons (PET) no estadiamento mediastinal e à distância do câncer pulmonar de células não pequenas.

**MÉTODOS:**

As estimativas foram calculadas pelo método epidemiológico para os anos de 2014 a 2018. Utilizaram-se dados nacionais de incidência; de distribuição de doença e acurácia das tecnologias procedentes da literatura, e de custos, de estudo de microcustos e das bases de dados do Sistema Único de Saúde. Analisaram-se duas estratégias de uso da PET: oferta a todos os pacientes recém-diagnosticados, e restrita àqueles que apresentassem resultados negativos de tomografia computadorizada (TC) prévia. Foram realizadas análises de sensibilidade univariadas e por cenários extremos para avaliar influência de fontes de incertezas nos parâmetros utilizados.

**RESULTADOS:**

A incorporação da PET-TC ao Sistema Único de Saúde implicaria na necessidade de recursos adicionais de R$158,1 (US$98,2) milhões para oferta restrita a R$202,7 (US$125,9) milhões para oferta abrangente em cinco anos, com diferença entre as duas estratégias de oferta de R$44,6 (US$27,7) milhões no período. Em termos absolutos, o impacto orçamentário total da incorporação no Sistema Único de Saúde, em cinco anos, seria de R$555 (US$345) e R$600 (US$372,8) milhões, respectivamente. Custos do procedimento PET-TC foram o parâmetro de maior influência nos resultados. No cenário por extremos mais otimista, os impactos orçamentários incrementais reduzir-se-iam para R$86,9 (US$54) e R$103,8 (US$64,5) milhões, considerando PET-TC para TC negativa e PET-TC para todos, respectivamente.

**CONCLUSÕES:**

A incorporação da PET no estadiamento clínico do câncer pulmonar de células não-pequenas parece ser financeiramente factível frente à magnitude do orçamento do Ministério da Saúde. A potencial redução no número de cirurgias desnecessárias pode produzir maior eficiência na alocação dos recursos disponíveis.

## INTRODUÇÃO

Avaliações econômicas sobre intervenções diagnósticas e terapêuticas são cada vez mais importantes para subsidiar decisões relativas à incorporação e difusão de novas tecnologias em saúde.^22^ Essas análises, contudo, não fornecem toda informação necessária à tomada de decisão, pois não avaliam a factibilidade da introdução da melhor alternativa frente aos orçamentos disponíveis.^11^ É necessária a realização adicional de análises de impacto orçamentário que avaliem as consequências financeiras, de curto e médio prazo, relacionadas à incorporação, alteração do uso ou retirada de uma tecnologia do conjunto de intervenções disponíveis no sistema de saúde.^2,8^

O câncer de pulmão é altamente incidente no Brasil: 27.330 novos casos estimados para 2014.^a^ Os carcinomas pulmonares de células não pequenas (CPCNP) correspondem a 75,0%-85,0% dos casos, com potencial de cura por ressecção cirúrgica na doença localizada.^5,^ O diagnóstico é muitas vezes realizado em estágios avançados. Assim, devido à extensão da doença para linfonodos mediastinais ou de metástases à distância, no momento do diagnóstico, apenas 20,0% dos pacientes são considerados operáveis.^c^

A avaliação da extensão da doença ao diagnóstico é essencial para a definição da terapêutica. Isso evita procedimentos inadequados que podem impactar na sobrevida e na qualidade de vida. O estadiamento clínico é feito principalmente por tomografia computadorizada com contraste de tórax e abdome superior (TC de tórax), segundo as diretrizes clínicas para o diagnóstico e tratamento do câncer de pulmão, divulgadas pelo Ministério da Saúde (MS), em 2012.^d^ Esse exame baseia-se, sobretudo, em alterações morfológicas.

A tomografia de emissão de pósitrons (PET), combinada à tomografia computadorizada (PET-TC) ou não, tem base em atividade metabólica e não apenas em aspectos anatômicos. Ambas possuem acurácia superior às técnicas de imagem convencionais na avaliação do comprometimento mediastinal e à distância.^5^ Sua adição às estratégias diagnósticas tradicionais pode resultar em melhor manuseio dos casos, com redução do número de cirurgias desnecessárias^21,23^ e da morbimortalidade associada. Outra vantagem seria o estadiamento da doença torácica e à distância a partir de um único exame.^14^

A PET encontra-se em fase incipiente de difusão no Brasil e foi incorporada às tabelas de pagamento de procedimentos do Sistema Único de Saúde (SUS) em abril de 2014.^e^ Avaliação econômica do uso da PET-TC no estadiamento do CPCNP, realizada para o MS em 2013, indicou que a PET-TC apresenta melhor custo-efetividade em comparação à estratégia de manejo atualmente oferecida, baseada em TC.^b^ Os resultados confirmam achados internacionais,^4^ que mostram benefícios da sua inclusão no estadiamento dos pacientes com CPCNP, sobretudo na prevenção de cirurgias desnecessárias, que compensam o custo adicional do uso da tecnologia.

O estudo de 2013 não avaliou os impactos financeiros da oferta do procedimento na rede de serviços públicos de saúde. Avaliações de impacto orçamentário são escassas no País, sobretudo na área de diagnóstico por imagem. Em um sistema de saúde que se propõe ofertar cuidado universal e integral, a preocupação com o uso dos recursos mostra-se importante frente à relação dicotômica entre disponibilidade orçamentária, expansão do cuidado e contínuo surgimento de novas tecnologias.

Este trabalho teve como objetivo estimar o impacto orçamentário da incorporação da PET-TC no estadiamento mediastinal e à distância do câncer pulmonar de células não pequenas.

## MÉTODOS

A estimativa do impacto orçamentário adotou a perspectiva do SUS como financiador dos serviços de saúde, como indicado pela diretriz brasileira.^f^

O horizonte escolhido foi de cinco anos (2014 a 2018), considerando a possível lentidão na realocação dos orçamentos governamentais e restrições na disponibilidade e acesso à PET-TC.

As projeções de utilização da PET-TC foram feitas pelo método epidemiológico. Pacientes elegíveis ao uso correspondem a todos os casos recém-diagnosticados. Assim, utilizaram-se estimativas de incidência de câncer pulmonar para 2006 a 2014 divulgadas pelo Instituto Nacional de Câncer (Tabela 1), com 85,0% do total de casos atribuídos ao tipo histológico CPCNP.^5^ Os novos casos foram estimados admitindo cobertura de 75,0% da população atendida pelo SUS.^g^

**Tabela 1:** Incidência de câncer de pulmão e do CPCNP, 2006 a 2014, e projeção do número de casos novos de CPCNP, 2006 a 2014, e atendidos pelo Sistema Único de Saúde, 2014 a 2018.

Ano	Casos novos câncer de pulmão	Casos Novos CPCNP	Casos Novos CPCNP atendidos pelo SUS
2006	27.170	23.095	–
2007	27.170	23.095	–
2008	27.270	23.180	–
2009	27.270	23.180	–
2010	27.630	23.486	–
2011	27.630	23.486	–
2012	27.320	23.222	–
2013	27.320	23.222	–
2014^a^	27.330	23.231	17.423
2015^b^	–	23.248	17.436
2016^b^	–	23.266	17.449
2017^b^	–	23.283	17.462
2018^b^	–	23.301	17.475

Fonte: Estimativas a partir dos dados de incidência de câncer divulgados pelo Instituto Nacional de Câncer em 2005, 2007, 2009, 2011 e 2014.

CPCNP: Câncer pulmonar de células não pequenas; SUS: Sistema Único de Saúde

a Foram repetidos os dados preliminares da fonte: Ministério da Saúde. Instituto Nacional de Câncer. Estimativa 2014: incidência de câncer no Brasil. Rio de Janeiro (RJ); 2014.

b Estimativa a partir da variação dos anos 2006 a 2014.

Foram definidos três cenários de análise: de referência (estratégias de manejo atualmente utilizadas baseada em TC de tórax a todos os pacientes); alternativo 1 (uso da PET-TC restrito a pacientes com resultados de TC prévia negativos, permitindo contemplar situações de acesso mais limitado à PET-TC); alternativo 2 (utilização de TC e PET-TC para todos os casos, com manuseio clínico posterior definido pelos resultados combinados dos dois exames – apenas pacientes com ambas as imagens negativas procederiam diretamente para ressecção pulmonar). Esta última estratégia representou maior redução das cirurgias desnecessárias no estudo de custo-efetividade de base,^b^ com diferenças pequenas na razão de custo-efetividade incremental entre as duas formas de uso da PET-TC nas análises de sensibilidade realizadas.

Foram considerados apenas os custos diretos de procedimentos envolvidos no estadiamento e terapêutica dos pacientes (Tabela 2). Como o procedimento PET-TC não se encontrava incorporado às tabelas de pagamento do SUS quando da realização das análises, utilizaram-se valores estimados por microcustos.^3^ Os valores foram recalculados com redução de 30,0% nos custos do F^18^-fluoro-2-desoxi-D-glicose (^18^FDG)^h^ para contemplar a recente expansão dos produtores privados do insumo advinda da queda do monopólio da União na produção de radiofármacos em 2006. Para todos os procedimentos presentes nas tabelas do SUS, foram utilizados valores de novembro de 2013, presentes no Sistema de Gerenciamento da Tabela de Procedimentos, Medicamentos e Órteses, Próteses e Materiais Especiais do SUS.^i^

**Tabela 2:** Parâmetros de custos, acurácia e epidemiológicos utilizados na análise de impacto orçamentário e fontes de dados.

Parâmetro	Valor	Intervalo	Referências
Custos			
TC de tórax (R$)	136,41	–	Sigtap/Datasus^i^
PET-TC de corpo inteiro (R$)	2.676,76	1.637,87;2.927,19	Caetano^3^ (2014) + Pressupostos
Mediastinoscopia (R$)	1.385,19	–	Sigtap/Datasus^i^
Biópsia (R$)	963,87	–	Sigtap/Datasus^i^
Cirurgia (R$)	4.326,58	–	Sigtap/Datasus^i^
Quimioterapia + Radioterapia (R$)	3.890,00	–	Sigtap/Datasus^i^
Tratamento paliativo (R$)	1.100,00	–	Sigtap/Datasus^i^
Morte por mediastinoscopia (R$)	2.717,31	–	Sigtap/Datasus^i^
Acurácia			
Sensibilidade TC de tórax para linfonodos mediastinais (%)	51	47;62	Dwamena^7^ (1999); Silvestri^18^ (2007)
Sensibilidade biópsia para metástases à distância (%)	100	80;100	Gambhir^9^ (1996); Sloka^19^ (2004)

Parâmetros epidemiológicos			

Taxa de variação da incidência (%)	0,0754	-1,1983;0,4054	Estimativas do INCA (2005, 2007, 2009, 2011, 2014)
Prevalência de metástases à distância (%)	20	12;25	NICE 2011^c^
Prevalência de metástases em linfonodos mediastinais (%)	30	15;40	Dietlein^6^ (2000); NICE 2011^c^
Probabilidade de realização de mediastinoscopia (%)	50	0;100	Alzahouri^1^ (2005) consulta especialistas

TC: Tomografia computadorizada; INCA: Instituto Nacional de Câncer; PET-TC: Tomografia de emissão de pósitrons conjugada à tomografia computadorizada

Para as estimativas de impacto orçamentário, foram utilizadas as mesmas árvores de decisão e parâmetros empregados no estudo de custo-efetividade realizado para o MS em 2013.^b^ Os casos novos projetados por ano e os custos dos procedimentos alimentaram as árvores relacionadas a cada cenário de análise, gerando estimativas de quantidades de procedimentos realizados e custos totais associados àquela população-alvo. Foram calculados os impactos orçamentários anuais e para o período 2014-2018, para cada cenário. Não foram introduzidos desconto ou ajustes inflacionários, atendendo às diretrizes internacionais^1,2,13,16^ e nacional^f^ sobre este tipo de estudo.

O impacto orçamentário incremental para cada ano examinado foi calculado pela diferença entre os impactos orçamentários totais dos cenários alternativos e o de referência. A diferença incremental entre as estratégias alternativas foi avaliada, permitindo a análise entre oferta mais ampla e mais restrita da tecnologia.

Foram realizadas análises de sensibilidade univariadas e por “cenários extremos”, visando a contemplar as incertezas relacionadas aos valores de parâmetros e pressupostos utilizados.^15^ Os parâmetros avaliados nas primeiras foram: a taxa de variação anual da incidência de câncer pulmonar; custos do procedimento PET-TC; prevalência de lesões mediastinais e à distância; probabilidade de realização de mediastinoscopia confirmatória; e sensibilidade da TC e da PET-TC. Foram utilizadas as mesmas faixas de valores obtidas da literatura e empregadas no estudo para o MS.^b^

Os parâmetros foram modificados simultaneamente na análise de sensibilidade por “cenários extremos”. O “cenário mais otimista” correspondeu à minimização do impacto orçamentário da incorporação da PET para qualquer cenário alternativo adotado. Foram empregados os valores mínimos do intervalo disposto na Tabela 2 para os parâmetros: custo da PET-TC, taxa de variação anual da incidência e sensibilidade da TC. Simultaneamente, foram empregados em seus valores máximos: sensibilidade da biópsia; proporção de pacientes que realizavam mediastinoscopia; e prevalências de metástases em linfonodos mediastinais (N2/3) e à distância (M^1^). O “cenário mais pessimista” correspondeu aos mesmos parâmetros variados em sentido oposto ao mencionado acima.

Adicionalmente, foi analisada a influência da taxa de difusão da tecnologia no SUS. É possível que, mesmo incorporada às tabelas de pagamento do SUS, possa ocorrer atraso até sua plena oferta em virtude da atual disponibilidade geográfica de equipamentos e recursos humanos qualificados para seu manuseio. Considerou-se acesso dos pacientes candidatos ao uso da PET-TC de 60,0% em 2014 e incrementos anuais de 10,0%, atingindo acesso pleno em 2018.

Obteve-se autorização escrita junto à coordenadora do Projeto (CNPq 564797/2010-3) para uso dos dados e do modelo do estudo de custo-efetividade.

## RESULTADOS

O atual manejo diagnóstico-terapêutico dos pacientes com CPCNP nos serviços de saúde brasileiros, centrado no uso da TC, resultaria em gastos de R$397,5 (US$246,9)^j^ milhões em cinco anos para o SUS.

A introdução da PET-TC no estadiamento do CPCNP implicaria elevação dos gastos totais do SUS (Tabela 3) em virtude de seu caráter complementar e não substitutivo, independentemente da estratégia de uso. Seu uso restrito aos pacientes com resultado de TC de tórax negativo determinaria impacto total de R$555,5 (US$345,0) milhões no período (+39,8% em relação ao manuseio atual). Sua utilização em todos os pacientes produziria impacto de R$600,1 (US$372,8) milhões (+51,0%).

**Tabela 3:** Impacto orçamentário total e incremental por ano e para 2014 a 2018, relativos aos cenários de análise estudados (em Reais de 2013).

Período	Impacto orçamentário total			Impacto orçamentário incremental		

	TC	PET-TC para TC-^a^	PET-TC para todos^b^	PET-TC para TC-^a^ em relação à TC	PET-TC para todos^b^ em relação à TC	PET-TC para todos^b^ em relação à PET-TC para TC-^a^
2014	79.369.310,57	110.932.404,67	119.846.591,61	31.563.094,10	40.477.281,04	8.914.186,94
2015	79.429.117,27	111.015.994,92	119.936.898,92	31.586.877,65	40.507.781,65	8.920.903,99
2016	79.488.969,03	111.099.648,16	120.027.274,27	31.610.679,13	40.538.305,24	8.927.626,11
2017	79.548.865,89	111.183.364,44	120.117.717,73	31.634.498,54	40.568.851,83	8.934.353,29
2018	79.608.807,89	111.267.143,79	120.208.229,33	31.658.335,90	40.599.421,45	8.941.085,54
2014-2018	397.445.070,65	555.498.555,98	600.136.711,86	158.053.485,33	202.691.641,21	44.638.155,87

TC: Tomografia computadorizada; PET-TC: Tomografia de emissão de pósitrons conjugada à tomografia computadorizada

a PET-TC para TC-: realização de PET-TC apenas para pacientes com resultado de TC negativa.

b PET-TC para todos: realização de PET-TC para todos os pacientes considerando ambos os resultados da PET e TC para prosseguimento do manejo clínico-terapêutico.

O impacto financeiro da oferta mais restrita da PET-TC implicaria alocação adicional de R$158 (US$98,2) milhões em cinco anos (Tabela 3). A extensão da oferta a todos os potenciais candidatos envolveria R$202,7 (US$125,9) milhões em recursos adicionais, com diferença entre as estratégias de R$44,6 (US$27,7) milhões ao final do período.

O custo do procedimento PET-TC foi o parâmetro de maior impacto nas análises de sensibilidade univariadas (Figura) utilizando os valores da faixa presentes na Tabela 2. Redução no custo do procedimento para R$1.637,87 (US$1.017,31) levaria à redução no impacto orçamentário total em cinco anos de R$67,9 (US$42,2) milhões na oferta restrita (-12,2%) e de R$90,6 (US$56,3) milhões no uso mais abrangente (-15,1%). A diferença entre as duas estratégias cairia para R$21,9 (US$13,6) milhões. Elevação do custo do procedimento para R$2.927,19 (US$1.818,13) resultaria em aumentos relativamente menores no impacto orçamentário total: R$16,4 (US$10,2) milhões (+2,9%) na oferta restrita e R$21,9 (US$13,6) milhões (+3,6%) na oferta abrangente.

**Figura:** Resultado da análise de sensibilidade univariada do impacto orçamentário total. Cenários avaliados, Brasil, 2014 a 2018. (Figura expressa em Reais)

A variação da proporção de pacientes submetidos à mediastinoscopia entre 0% e 100% para comprovação dos resultados dos exames de imagem mostrou-se importante, dado seus custos para o SUS. A não realização de mediastinoscopia correspondeu à redução no impacto orçamentário total de R$24,6 (US$15,3) milhões no cenário “PET-TC para TC-” e de R$20,3 (US$12,6) milhões no uso da “PET para todos”. Sua realização em todos os pacientes, por outro lado, levaria a acréscimos nos mesmos montantes nos dois cenários.

O uso do valor inferior da faixa de crescimento de casos novos de CPCNP elegíveis ao estadiamento produziu queda no impacto orçamentário, independentemente do cenário analisado: de R$14 (US$8,7) milhões, no cenário de “PET-TC para TC-”, e de R$15,1 (US$9,4) milhões, com oferta da “PET-TC para todos”. O uso do limite superior do parâmetro acarretou aumento de R$3,7 (US$2,3) milhões e R$4 (US$2,5) milhões, respectivamente.

As análises de sensibilidade por cenários extremos (Tabela 4) mostraram significativa redução no impacto orçamentário total no “cenário mais otimista”: R$90,3 (US$56,1) milhões na utilização abrangente da PET-TC (-15,0%), e R$62,5 (US$38,8) milhões na oferta restrita (-11,3%). A incorporação geraria aumentos do impacto orçamentário de R$25,9 (US$16,1) milhões na estratégia “PET-TC para todos” (+4,3%), e de R$22,4 (US$13,9) milhões no uso restrito (+4,0%) no “cenário mais pessimista”.

**Tabela 4:** Análise por cenários do impacto orçamentário total e incremental das estratégias de estadiamento por ano de projeção (em Reais de 2013). Brasil, 2014 a 2018.

Período	Impacto orçamentário total			Impacto orçamentário incremental		

	TC	PET para TC-^a^	PET-TC para todos^b^	PET para TC-^a^ em relação à TC	PET-TC para todos^b^ em relação à TC	PET-TC para todos^b^ em relação à PET para TC-^a^
Caso base						
2014	79.369.310,57	110.932.404,67	119.846.591,61	31.563.094,10	40.477.281,04	8.914.186,94
2015	79.429.117,27	111.015.994,92	119.936.898,92	31.586.877,65	40.507.781,65	8.920.903,99
2016	79.488.969,03	111.099.648,16	120.027.274,27	31.610.679,13	40.538.305,24	8.927.626,11
2017	79.548.865,89	111.183.364,44	120.117.717,73	31.634.498,54	40.568.851,83	8.934.353,29
2018	79.608.807,89	111.267.143,79	120.208.229,33	31.658.335,90	40.599.421,45	8.941.085,54
2014-2018	397.445.070,65	555.498.555,98	600.136.711,86	158.053.485,33	202.691.641,21	44.638.155,87
Cenário mais otimista^c^						
2014	83.187.682,93	100.981.815	104.449.358	17.794.132,39	21.261.675	3.467.543
2015	82.190.832,17	99.771.735	103.197.725	17.580.902,57	21.006.893	3.425.991
2016	81.205.926,81	98.576.155	101.961.091	17.370.227,92	20.755.164	3.384.937
2017	80.232.823,74	97.394.902	100.739.276	17.162.077,81	20.506.452	3.344.374
2018	79.271.381,50	96.227.803	99.532.102	16.956.422,00	20.260.720	3.304.298
2014-2018	406.088.647,15	492.952.409,83	509.879.552,34	86.863.762,68	103.790.905,19	16.927.142,51
Cenário mais pessimista^d^						
2014	76.145.605,34	114.636.179	124.202.351	38.490.573,19	48.056.746	9.566.172
2015	76.454.329,42	115.100.958	124.705.916	38.646.629,04	48.251.586	9.604.957
2016	76.764.305,19	115.567.623	125.211.522	38.803.317,59	48.447.217	9.643.900
2017	77.075.537,71	116.036.179	125.719.179	38.960.641,42	48.643.641	9.683.000
2018	77.388.032,10	116.506.635	126.228.894	39.118.603,11	48.840.862	9.722.258
2014-2018	383.827.809,77	577.847.574,11	626.067.861,70	194.019.764,35	242.240.051,93	48.220.287,58

TC: Tomografia computadorizada; PET-TC: Tomografia de emissão de pósitrons conjugada à tomografia computadorizada

a PET-TC para TC-: realização de PET-TC apenas para pacientes com resultado de TC negativa.

b PET-TC para todos: realização de PET-TC para todos os pacientes considerando ambos os resultados da PET e TC para prosseguimento do manejo.

c Cenário mais otimista – variação para limite inferior do intervalo dos parâmetros: custo da PET-TC, taxa de variação anual da incidência, e sensibilidade da TC; e para valores máximos dos parâmetros: sensibilidade da biópsia, proporção de pacientes que realizavam mediastinoscopia e prevalências de metástases em linfonodos mediastinais (N2/3) e à distância (M1).

d Cenário mais pessimista – variação para limite superior do intervalo dos parâmetros: custo da PET-TC, taxa de variação anual da incidência e sensibilidade da TC; e para valores mínimos dos parâmetros: sensibilidade da biópsia, proporção de pacientes que realizavam mediastinoscopia e prevalências de N2/3 e M1.

A redução no impacto orçamentário total seria de R$31,6 (US$19,6) milhões (-5,7%) no cenário de oferta restrita da tecnologia, e de R$44 (US$27,4) milhões com sua disponibilização para todos (-7,3%), considerando progressiva difusão – de 60,0% a 100% em cinco anos – da PET-TC no SUS.

## DISCUSSÃO

A incorporação da PET-TC ao SUS no estadiamento do CPCNP, neoplasia de grande relevância no cenário nosológico nacional, implicaria em gastos totais para o SUS de R$555,5 (US$345,0) milhões, no caso de seu uso restrito a pacientes com resultados prévios negativos à tomografia computadorizada de tórax, e de R$600,1 (US$372,8) milhões, em situação de oferta a todos os novos casos diagnosticados no período. Estes valores representam recursos adicionais aos atuais gastos com o estadiamento baseado na tomografia computadorizada, de cerca de R$158,1 (US$98,2) a R$202,7 (US$125,9) milhões em cinco anos, dependendo da sua utilização mais restrita ou abrangente. Em termos absolutos ou incrementais, os valores estimados reforçam a importância do planejamento e gestão adequados do orçamento e das ações governamentais, incluindo a assistência em saúde, de modo a aperfeiçoar o uso dos escassos recursos disponíveis em nosso meio.

A Política Nacional de Gestão de Tecnologia em Saúde^k^ e a Lei 12.401^l^ têm reconhecido o papel das avaliações econômicas complementares. A realização de estudos de impacto orçamentário como apoio às decisões de incorporação de novas tecnologias no SUS é recomendada explicitamente.

Um dos desafios do SUS repousa na obediência ao princípio da universalidade. Ofertar PET-TC a todos os candidatos pode ser inviável, devido a limitações financeiras, de infraestrutura ou de recursos humanos, entre outras. Esse reconhecimento e o fato de a literatura e o estudo do Ministério da Saúde apontarem maiores benefícios em saúde para o grupo com resultados negativos à TC prévia^6,^ levaram à simulação da oferta restrita do exame. Contudo, a expansão da oferta a todos os potenciais candidatos implicaria acréscimo ao impacto orçamentário total de apenas R$44,6 (US$27,7) milhões ao final do período.

A magnitude dos impactos associados à incorporação da PET-TC tem implicações financeiras significativas, sobretudo se ponderado o número de indivíduos elegíveis ao seu uso. Isso porque, em 2013, o número de casos novos de câncer pulmonar correspondeu a apenas 0,01% da população brasileira.

As despesas do Ministério da Saúde presentes na Lei Orçamentária Anual para 2014^m^ foram levantadas para melhor compreensão do significado dos volumes de recursos estimados com a incorporação da PET-TC. O valor estimado para o manuseio atual disponível no SUS corresponde a 0,075% dos R$106.019.264.465,00 (US$65.850.474.822,98) previstos para 2014. Nos cenários alternativos, o impacto orçamentário total estimado corresponderia a 0,105% do orçamento do MS (oferta restrita) ou a 0,113% (oferta abrangente).

Outra forma de examinar a representatividade dos impactos estimados seria compará-los aos valores gastos pelo SUS com assistência aos pacientes com câncer pulmonar, e.g., em termos de diagnóstico e tratamento, em suas várias modalidades. Entretanto, não foram encontradas informações consolidadas na literatura ou em documentos oficiais sobre os volumes de gastos com essa condição. Os gastos do Ministério da Saúde relacionados ao pagamento de exames de TC de tórax, realizados e aprovados no SUS, podem ser obtidos no Sistema de Informações Ambulatoriais,^n^ mas correspondem ao uso do procedimento em diversas indicações clínicas (neoplásicas e não neoplásicas), e não apenas para câncer pulmonar, o que impossibilita quaisquer comparações. Despesas apenas com internações do SUS relacionadas ao câncer de pulmão, obtidas no Sistema de Informações Hospitalares, somaram R$23.405.185,25 (US$14.537.382,14) de janeiro a novembro de 2013.^o^ Isso representa 29,5% dos R$79,4 (US$49,3) milhões do impacto orçamentário estimado no cenário de referência para 2014, mas não inclui os demais componentes diagnósticos e terapêuticos envolvidos no manuseio da condição.

A difusão da PET-TC para a prática clínica ocorreu em um contexto em que se avolumavam preocupações com gastos e impactos para os sistemas de saúde. Assim, a tecnologia foi objeto de múltiplos estudos de custo-efetividade em diversos países. Avaliações de impacto orçamentário de sua implementação são menos frequentes na literatura, talvez porque sejam realizadas internamente no meio governamental envolvido com a oferta da tecnologia. Entretanto, comparações diretas dos resultados dessas análises de impacto orçamentário com as apresentadas neste estudo são impróprias. Isso porque a gestão e organização dos sistemas de saúde, estruturas dos modelos, dados epidemiológicos e, sobretudo, as estruturas de custo subjacentes, são diversas entre si.^17^

Comparar estimativas de impacto orçamentário realizadas na nossa realidade seriam ideais. Embora o MS tenha simulado internamente o impacto orçamentário da PET no SUS,^p^ seus métodos de estimação e possibilidade de importantes vieses metodológicos dificultam comparações com os resultados deste estudo. Além disso, segundo as diretrizes de impacto orçamentário, as estimativas não devem ser restritas à comparação das quantidades e valores das tecnologias *per se*, mas ao resultado financeiro do conjunto de consequências clínicas e de procedimentos diagnóstico-terapêuticos relacionados às tecnologias examinadas, como se objetivou neste estudo.

Apesar de o emprego da PET não representar aumento significativo na sobrevida dos pacientes,^4^ seu uso permite melhor alocação de recursos (financeiros, materiais e humanos) no sistema, pois identifica mais acuradamente a extensão da doença e permite o planejamento da estratégia terapêutica mais adequada para cada caso. Esse melhor encaminhamento evitaria procedimentos cirúrgicos desnecessários, o que assume maior relevância quando há sabidamente problemas de acesso aos serviços de saúde no País, sobretudo oncológicos, além de significativas disparidades regionais na sua oferta.^10,^

Estudos de impacto orçamentário são escassos e apenas mais recentemente começaram a ter diretrizes de boas práticas estabelecidas. O presente trabalho seguiu as principais diretrizes disponíveis sobre análises de impacto orçamentário da *Task Force on Good Research Practices da International Society for Pharmacoeconomics and Outcomes Research* ^13^ e do Ministério da Saúde recentemente publicadas.^f^ Foram realizadas adaptações necessárias, já que estas focam principalmente procedimentos de natureza terapêutica.

Apesar de usarmos uma série temporal de nove anos (2006 a 2014) para projetar futuras incidências de câncer pulmonar, não foi possível prever eventuais alterações decorrentes de mudanças populacionais ou na prevalência de alguns de seus fatores de risco. Além disso, este trabalho utilizou valores de parâmetros oriundos do estudo de custo-efetividade. Dessa forma, permanecem as mesmas limitações lá presentes, como lacuna de dados nacionais relativos a alguns parâmetros epidemiológicos, medidas de acurácia das tecnologias diagnósticas oriundas de estudos internacionais, e desconhecimento da proporção de pacientes presentemente submetidos à mediastinoscopia no País. As múltiplas análises de sensibilidade realizadas buscaram minimizar essas incertezas e potencializar o conhecimento da ordem de impacto para o SUS, delas advindas.

Trueman et al^20^ discutem a incompatibilidade entre a busca pela maximização da eficiência, frequentemente objetivo central dos economistas, e os limites dos orçamentos anuais, comumente a principal necessidade dos gestores. Análises de impacto orçamentário não mostram a melhor forma de alocação dos recursos disponíveis na economia, cujas evidências mais adequadas provêm dos estudos de avaliação econômica completa, como os de custo-efetividade. Ademais, as decisões de incorporação de tecnologias nos sistemas de saúde devem considerar outros fatores, como a disponibilidade de recursos humanos e orçamentários, fatores políticos e aspectos relativos à equidade de acesso.

Dados similares aos deste estudo, aliados às evidências de custo-efetividade da tecnologia no Brasil, podem permitir maior embasamento nas decisões tomadas. Assim, a incorporação da PET-TC no estadiamento clínico do CPCNP, potencialmente ressecável, parece ser financeiramente factível frente à magnitude do orçamento total do MS e a potencial redução de cirurgias desnecessárias realizadas em pacientes melhor estadiados. Isso pode trazer maior eficiência na alocação dos recursos disponíveis.

## Footnotes

a Ministério da Saúde. Instituto Nacional de Câncer. Estimativa 2014: incidência de câncer no Brasil. Rio de Janeiro (RJ); 2014.

b Caetano R, Biz AN, Bastos CRG, Garay OU, Schluckebier L. Avaliação econômica: análise de custo-efetividade do uso da 18FDG-PET/TC no estadiamento do câncer pulmonar de células não pequenas: relatório preliminar de pesquisa. Rio de Janeiro (RJ): Instituto de Medicina Social da UERJ; 2013.

c National Collaborating Centre for Cancer. Lung cancer. The diagnosis and treatment of lung cancer. London: NICE; 2011 [citado 2012 out 10] (Nice Clinical Guidelines; 121). Disponível em: http://www.ncbi.nlm.nih.gov/books/NBK99021/

d Ministério da Saúde. Portaria nº 600, de 26 de junho de 2012. Aprova as diretrizes diagnósticas e terapêuticas do câncer de pulmão. *Diário Oficial União. *28 Jun 2012;Seção1:210.

e Ministério da Saúde. Portaria nº 7, de 22 de abril de 2014. Torna pública a decisão de incorporar o PET-CT no estadiamento clínico do câncer de pulmão de células não-pequenas potencialmente ressecável no Sistema Único de Saúde - SUS. *Diário Oficial União. *23 Abr 2014;Seção1:78.

f Ministério da Saúde. Secretaria de Ciência, Tecnologia e Insumos Estratégicos. Diretrizes metodológicas: análise de impacto orçamentário: manual para o Sistema de Saúde do Brasil. Brasília (DF); 2012.

g Agência Nacional de Saúde Suplementar. Caderno de informação da Saúde Suplementar: beneficiários, operadoras e planos. Rio de Janeiro (RJ); 2013.

h Ministério da Ciência, Tecnologia e Inovação. Comissão Nacional de Energia Nuclear (CNEN). Relatório de Gestão do Exercício de 2011. Rio de Janeiro (RJ); 2012.

i Ministério da Saúde. Departamento de Informática do SUS (DATASUS). Sistema de Gerenciamento da Tabela de Procedimentos, Medicamentos e OPM do SUS (SIGTAP). Brasília (DF); 2013 [citado 2013 nov 25]. Disponível em: http://sigtap.datasus.gov.br/tabela-unificada/app/sec/inicio.jsp

j A conversão de todos os valores monetários para dólar apresentados no texto, nas tabelas e figura, foi realizada a partir do fator de conversão PPP para o Brasil, ano de 2013, divulgado pelo Banco Mundial/ World Bank. PPP conversion factor, GDP (LCU per international $). Washington (DC): World Bank; 2015 [citado 2015 mai 2]. Disponível em: http://data.worldbank.org/indicator/PA.NUS.PPP

k Ministério da Saúde. Secretaria de Ciência, Tecnologia e Insumos Estratégicos. Política de Gestão de Tecnologias em saúde. Brasília (DF); 2011.

l Brasil. Lei nº 12.401, de 28 de abril de 2011. Altera a Lei nº 8.080, de 19 de setembro de 1990, para dispor sobre a assistência terapêutica e incorporação de tecnologias em saúde no âmbito do SUS. *Diário Oficial União*. 29 Abr 2011:Seção1:1-2

m Senado Federal. Portal do Orçamento. Lei Orçamentária Anual 2014: Volume IV – Detalhamento das Ações – Órgão do Poder Executivo – Presidência da República e Ministérios (exceto MEC). 36000. Brasília (DF); 2014 [citado 2014 jan 13]. Disponível em: http://www12.senado.gov.br/orcamento/loa?ano=2014&categoria=3.1.7&fase=elaboracao

n Ministério da Saúde. Departamento de Informática do SUS (DATASUS). Sistema de Informações Ambulatoriais do SUS (SIA/SUS). Brasília (DF); 2014. Disponível em: http://www2.datasus.gov.br/DATASUS/index.php?area=0202

o Ministério da Saúde. Departamento de Informática do SUS (DATASUS). Sistema de Informações Hospitalares do SUS (SIH/SUS). Brasília (DF); 2014 [citado 2014 Jan 15]. Disponível em: http://www2.datasus.gov.br/DATASUS/index.php?area=0202

p Ministério da Saúde. Secretaria de Ciência, Tecnologia e Insumos Estratégicos. PET-TC no estadiamento de câncer pulmonar de células não-pequenas. Relatório de Recomendação da Comissão Nacional de Incorporação de Tecnologias no SUS – CONITEC-107. Brasília (DF); 2014. Disponível em: http://portalsaude.saude.gov.br/images/pdf/2014/abril/23/Relatorio-PET-EstadiamentoCancerPulmonar-FINAL.pdf

## DESTAQUES

Apenas 20,0% dos pacientes com câncer de células não pequenas (CPCNP) são candidatos à cirurgia potencialmente curativa, no momento do diagnóstico. O correto estadiamento é fundamental para a escolha adequada do tratamento e existem evidências de que a tomografia de emissão de pósitrons (PET-TC) é uma ferramenta diagnóstica útil na determinação da extensão da doença.

Estudo brasileiro mostrou que a adição da PET-TC às técnicas de imagem convencionais é custo-efetiva para esta indicação. O procedimento foi incorporado às tabelas do Sistema Único de Saúde (SUS) em abril de 2014, mas ainda se desconhecem seus impactos econômico-financeiros.

Oferecer uma nova tecnologia de alto custo, como é o caso da PET-TC, gera gastos expressivos, tornando necessária a avaliação criteriosa do impacto para o SUS decorrente da disponibilização do procedimento.

A introdução da PET-TC no estadiamento do CPCNP implicaria na necessidade de recursos adicionais em relação ao atual manejo diagnóstico-terapêutico dos pacientes de R$158,1 milhões (oferta restrita aos casos com TC de tórax prévia negativa) a R$202,7 milhões (oferta abrangente a todos os pacientes recém-diagnosticados) ao final de cinco anos.

O custo do procedimento PET-TC foi o parâmetro de maior impacto nos resultados e reduções deste custo poderiam levar a diminuições expressivas nos valores do impacto orçamentário.

A incorporação da PET-TC no estadiamento do CPCNP parece ser financeiramente factível. Uma identificação mais acurada da extensão da doença e melhor planejamento do tratamento decorrentes do emprego da nova tecnologia poderiam evitar procedimentos cirúrgicos desnecessários, fato relevante quando se considera os problemas de acesso aos serviços de saúde oncológicos e as significativas disparidades regionais na sua oferta no País.

Rita de Cássia Barradas Barata

Editora Científica
